# Solid-State Thermal Decomposition in a Cu-Rich Cu-Ti-Zr Alloy

**DOI:** 10.3390/ma18133042

**Published:** 2025-06-26

**Authors:** Chenying Shi, Biaobiao Yang, Yuling Liu, Wei Shao, Yidi Li, Yunping Li, Dewen Zeng, Yong Du

**Affiliations:** 1 College of Chemistry and Chemical Engineering, Central South University, Changsha 410083, China; shichenying5@163.com (C.S.); dewen_zeng@hotmail.com (D.Z.); 2 IMDEA Materials Institute, C/Eric Kandel 2, Getafe, 28906 Madrid, Spain; biaobiao.yang@imdea.org (B.Y.); wei.shao@pw.edu.pl (W.S.); 3 Department of Materials Science, Polytechnic University of Madrid/Universidad Politécnica de Madrid, E.T.S. de Ingenieros de Caminos, 28040 Madrid, Spain; 4 State Key Laboratory of Powder Metallurgy, Central South University, Changsha 410083, China; liiidi@csu.edu.cn (Y.L.); lyping@csu.edu.cn (Y.L.)

**Keywords:** Cu-Ti-Zr alloy, solid-state decomposition, thermal stability, first principle calculations

## Abstract

Solid-state thermal decomposition in the Cu-13.3Ti-3.8Zr (at.%) alloy was studied using a synthesized method, including the temperature–concentration gradient and differential scanning calorimetry experiments within a single experimental cycle, as well as first principle calculations. Experimentally, the decomposition pathway and the solid solubility of Ti/Zr in the Cu matrix in the temperature range of 820 °C to 801.5 °C were observed in the Cu-13.3Ti-3.8Zr (at.%) alloy. The primary solid phase is (Cu) phase and subsequently precipitated Cu_51_Zr_14_ and Cu_4_Ti phases. These features are valuable for understanding the thermal stability and solid-state phase equilibria of the alloy. First principle calculations, including formation enthalpy, charge density, and electron localization function analyses, were conducted to evaluate the thermal, structural, and electrical stability of Cu_51_Zr_14_ with and without Ti doping, as well as Cu_4_Ti. The present work introduces an effective strategy for determining both the solid-state thermal decomposition pathway and the phase diagram within the solid-state region within a single experimental cycle.

## 1. Introduction

Copper (Cu) and its alloys are widely used in electrical, thermal, and structural applications due to their excellent electrical and thermal conductivities, good corrosion resistance, and adequate mechanical strength [1,2,3,4,5,6]. Among these, due to their high elasticity, fatigue resistance, and excellent electrical conductivity and ductility, Cu-Ti alloys are promising in applications such as electrical contact components for smartphones, computers, and automobiles. Thus, the age hardening of Cu-Ti alloys, which is a key behavior influencing their mechanical and electrical properties, has received extensive attention [7,8]. To further improve the strength and electrical conductivity of Cu-Ti-based alloys, the main strengthening strategies can be categorized as follows: manufacturing process optimization involving the powder metallurgy method [9,10], thermo-mechanical treatment optimization [7,11,12] (i.e., cold or hot deformation, aging treatment, and temperature), and microalloying with other metallic elements like B [13,14], Cr [15,16], and Zr [17,18,19]. Among these methods, microalloying is both economical and effective, with Zr being a commonly used alloying element due to its ability to significantly enhance mechanical properties by refining the microstructure, promoting the dispersion strengthening of precipitates [19,20]. To facilitate the determination of phase compositions and support the design of Cu-Ti-Zr alloys, an accurate solid-state region in the Cu-rich region of the Cu-Ti-Zr system is critically important. The types and stability of precipitates directly influence the mechanical strength and electrical conductivity of copper alloys. For example, the coherent β′-Cu_4_Ti precipitate can significantly enhance the mechanical properties of the Cu matrix through spinodal decomposition, although this slightly decreases its electrical conductivity [17,21,22]. Moreover, understanding these phase equilibria helps avoid undesirable phase transformations during service, thus improving the reliability of electrical contact components.

So far, the Cu-Ti-Zr system has been experimentally studied through isothermal sections from 703 °C to 800 °C by analyzing equilibrium alloys [23,24,25,26,27,28,29,30,31,32], with predictions of liquidus and solidus projections by means of differential thermal analysis (DTA) [31,32]. A thermodynamic description of the Cu-Ti-Zr system has been established using calculations according to the phase diagram (CALPHAD) method by Arroyave et al. [33] and Hermana et. al [23]. Traditional methods [34] for predicting phase diagrams—such as the phase-equilibrated alloy method—typically require prolonged annealing (e.g., 1440 h at 703 °C by Hermana et al. [23] or 760 h at 703 °C by Woychik and Massalski [26]) before combining the annealed and as-cast alloys with DTA to determine the solidification sequence. Although effective, these traditional methods are time-consuming. To address the limitations of traditional methods, this work introduces an experimental temperature–concentration gradient method that significantly reduces the experimental time, allowing for a more efficient determination of the solid-state thermal decomposition pathway. The principle of this method is to determine the solid-state decomposition pathway in the alloy within a single experimental cycle by annealing it under a controlled temperature–concentration gradient from low to high temperatures, with significantly less time required than traditional methods. First principle calculations based on density functional theory (DFT) have proven to be effective in revealing the thermodynamic stability and electronic structure of Cu-based alloys. Ghosh [35] systematically studied the energetics of Cu-Ti and Cu-Zr intermetallics, while Zhu et al. [36] and Tian et al. [37] further investigated their elastic and electronic properties. A recent work by Fotopoulos et al. [38] demonstrated the strengthening effect of Ti in Cu matrices through atomic-scale modeling. However, there are no DFT studies on the compounds in the Cu-Ti-Zr system. This work incorporates first principle calculations to complement experimental findings and provide a comprehensive analysis of the phase stability and decomposition mechanisms of the Cu-Ti-Zr alloy.

## 2. Materials and Methodology

### 2.1. Experimental Procedure

High-purity Cu (99.9 wt.%), Ti (99.9 wt.%), and Zr (99.9 wt.%) were used to prepare binary alloys. Then, Cu-20Zr (at.%) and Cu-25Ti (at.%) intermediate alloys were prepared by these pure alloys using a non-consumable electric arc furnace (WKDHL-1, Opto-electronics Co., Ltd., China) under an Ar atmosphere. Furthermore, a 60 g alloy sample with a target composition of Cu-3.8Zr-13.3Ti (at.%) was prepared by melting pure Cu (99.9 wt.%) and the alloys Cu-20Zr (at.%) and Cu-25Ti (at.%) in an induction furnace at 1200 °C with stirring, followed by an isothermal hold for 10 min to achieve a homogeneous liquid state. The molten alloy was then poured into a cylindrical graphite crucible with a diameter of 5 mm and a length of 250 mm to form a rod, followed by rapid quenching in water. Both ends of the rod sample were trimmed, and the middle section was polished with sandpaper to remove oxide layer. Next, the sample composition was analyzed using an inductively coupled plasma optical emission spectrometer (ICP-OES, Spectro Blue, Spectro Analytical Instruments GmbH, Kleve, Germany) to ensure accuracy. Then, one end of the polished rod was ground to a 60° conical tip, while the other end was flattened. Finally, the rod sample was prepared with a diameter of 5 mm and a length of 135 mm. 

**Figure 1:**
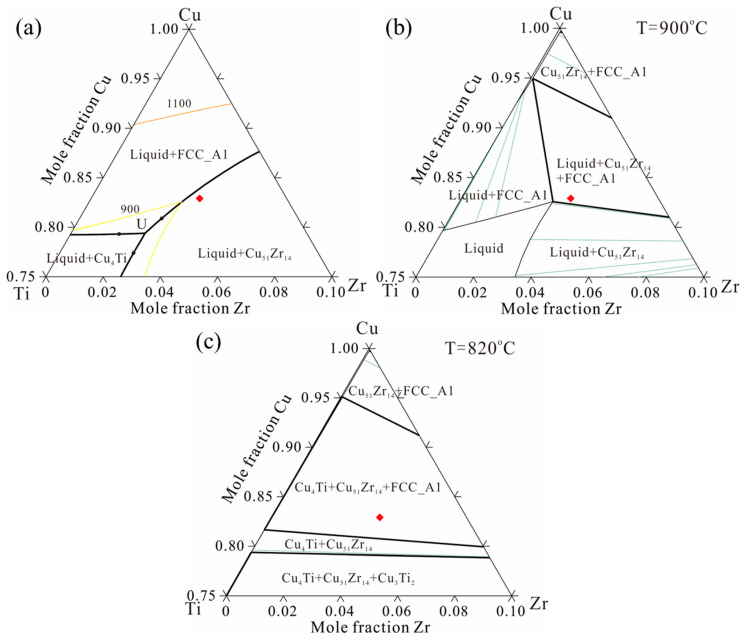
Preliminary phase diagram of the Cu-rich corner of the Cu-Ti-Zr system based on the thermodynamic parameters in Hermana et al. [23]: (**a**) liquidus projection, (**b**) isothermal section at 900 °C, (**c**) isothermal section at 820 °C (the red dot represents sample composition location).

**Figure 2:**
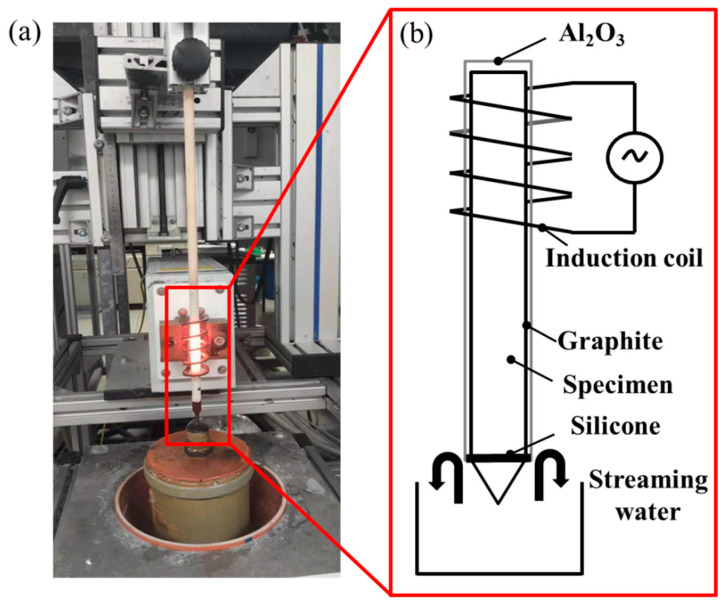
Experimental setup: (**a**) apparatus and (**b**) schematic diagram.

Based on the thermodynamic parameters from Hermana et al. [23], the predicted liquidus projection, isothermal section at 900 °C, and isothermal section at 820 °C are presented in Figure 1a–c, with the sample composition of Cu-13.3Ti-3.8Zr (at.%) marked by a red square. Figure 2 presents the equipment (Figure 2a) and a corresponding schematic diagram (Figure 2b). The rod sample was wrapped in graphite and placed inside an Al_2_O_3_; tubular crucible with an inner diameter of 6 mm. The tubular crucible was then positioned inside an induction coil measuring 45 mm in length and 10 mm in inner diameter. The temperature between the second and third windings of the induction coil was set to 820 °C, which is lower than the liquidus assessment from the thermodynamic parameters simulated by Hermana et al. [23]. The tip of the lower part of the rod sample was placed in flowing cooling water at 20 °C. The distance between the cooled tip in the water and the heated section of the second and third windings of the induction coil is 75 mm, with a temperature difference of 800 °C (820 °C − 20 °C). Consequently, the temperature gradient can be estimated as 800 °C/75 mm ≈ 10.7 °C/mm. After being held for 20 h, the phases in the alloy coarsened along the temperature gradient, forming distinct phase regions according to the solidification sequence. Subsequently, the rod sample was quickly quenched in cold water. The longitudinal cross-section of the sample was polished with sandpaper (320 #, 600 #, 1200 #, and 2000 #) and diamond suspensions (3 μm and 1 μm), followed by microstructural characterization using optical microscope (OM, DM4M, Leica, Wetzlar, Germany) and electron probe micro-analyzer (EPMA, JXA-8100, JEOL, Japan). A segment of approximately 3 mm was cut from a point 5 mm away from the tip, and after polishing the surface, 0.1 g of powder was ground from the segment for differential scanning calorimetry measurements (DSC, 404C, Netzsch, Germany) to determine the phase transition temperatures, and 0.5 g of powder was ground for X-ray diffractometer (XRD, Bruker-AXS D8, Germany) analysis to identify the phase types. The DSC test was conducted in Al_2_O_3_ crucibles with a heating and cooling rate of 15 °C/min between 25 °C and 750 °C and 3 °C/min between 760 °C and 1000 °C. 

### 2.2. DFT Calculations

The formation enthalpies of Cu_51_Zr_14_ with and without Ti doping as well as Cu_4_Ti were evaluated using density functional theory (DFT) calculations. A similar method has also been applied to Mg-based alloys and shows good agreement with experimental results [39]. The crystal system of Cu_51_Zr_14_ is hexagonal, with a space group of *P6*, and the configuration contains 65 atoms [40]. Cu_4_Ti has an orthorhombic crystal structure with space group *Pnma* and a unit cell containing 20 atoms [40]. In this work, DFT calculations were performed using the projector augmented-wave (PAW) method implemented in VASP [41,42,43,44,45,46]. Exchange and correlation functionals were treated using the generalized gradient approximation method (GGA-PBE) [47]. Total energies were calculated using the Monkhorst–Pack mesh [48] of k-points in the Brillouin zone, with k-mesh spacing of 0.02 Å^−1^. A cut-off energy of 450 eV was used for the plane wave basis in all of the calculations. The total energy convergence criterion was set to 10^−5^ eV/cell, and force components were relaxed to 10^−3^ eV/Å. 

## 3. Results and Discussion

### 3.1. Experimental Results

**Figure 3:**
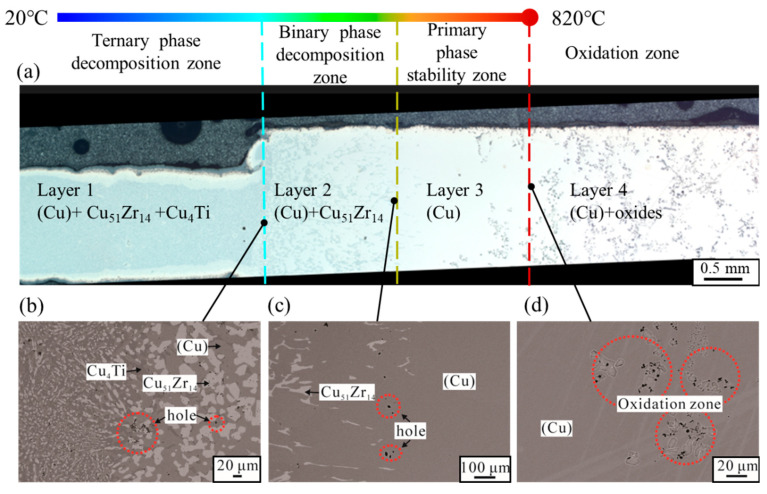
Details of the microstructure in the Cu-13.3Ti-3.8Zr (at.%) alloy after 20 h heat treatment under temperature gradient: (**a**) the optical micrograph; (**b**–**d**) microstructures at the interfaces of Layer 1/Layer 2, Layer 2/Layer 3, and Layer 3/Layer 4, observed in backscattered electron (BSE) mode.

**Figure 4:**
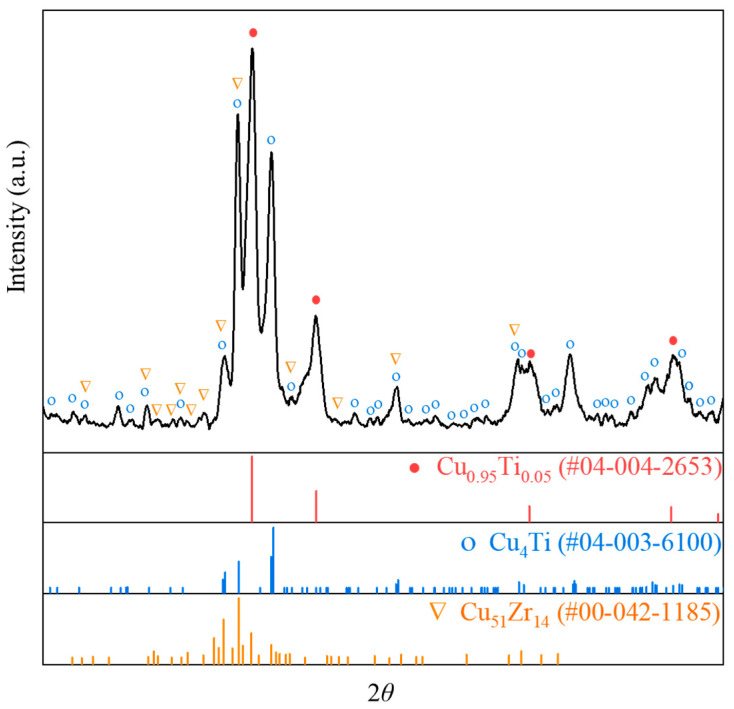
XRD results for the Cu-13.3Ti-3.8Zr (at.%) alloy in as-cast region.

The Cu-13.3Ti-3.8Zr (at.%) alloy was processed using the temperature–concentration gradient method described in Section 2.1. The optical micrograph of the rod sample after 20 h is shown in Figure 3a. The microstructures at the interfaces of Layer 1/Layer 2, Layer 2/Layer 3, and Layer 3/Layer 4, observed in backscattered electron (BSE) mode using EPMA, are presented in Figure 3b–d. The region of the rod sample near 20 °C remains almost in a cast condition, and the XRD pattern of this region is displayed in Figure 4. The XRD patterns of PDF cards #04–004–2653 (Cu_0.95_Ti_0.05_), #04–003–6100 (Cu_4_Ti), and #00–042–1185 (Cu_51_Zr_14_) are depicted. Note that the Cu_0.95_Ti_0.05_ phase can be regarded as the (Cu) phase. Based on the as-cast microstructure observed in the left region of Figure 3b, the composition comprises a primary phase (Cu) in grey, and a eutectic mixture of Cu_51_Zr_14_ + Cu_4_Ti. To minimize the effect of the surface oxide layer on the composition analysis, the compositions of the rod core were determined using EPMA, and the constituent phases and their corresponding compositions in the regions shown in Figure 3b–d are summarized in Table 1. Noted that the uncertainties (±) in Table 1 indicate the 95% confidence intervals (CI_95%_) from multiple measurements, and the ranges indicate composition fluctuations across inhomogeneous regions. And CI_95%_ is expressed as follows:

(1) $${\mathrm{CI}}_{95\%}=\bar{x}\pm t_{0.025,n-1}\cdot \frac{s}{\sqrt{n}}$$

where $\bar{x}$ is the average value, s is the sample standard deviation, n is the sample size, and $t_{0.025,n-1}$ is the critical value for a 95% confidence level.

Based on the XRD and EPMA results, the phases in the low-temperature region identified in the Cu-13.3Ti-3.8Zr (at.%) alloy are (Cu), Cu_4_Ti, and Cu_51_Zr_14_, which are consistent with those reported in the literature for alloys of similar compositions, such as alloy #11 (Cu-10.0Ti-4.0Zr, at.%) aged at 703 °C for 1440 h in Hermana et al. [23] and alloy #63 (Cu-13.5Ti-3.5Zr, at.%) aged at 750 °C for 536 h in Storchak et al. [31]. As shown in Figure 3, the solid-phase composition of the rod sample from high temperature to low temperature (Layer 4 to Layer 1) is as follows: Oxides + (Cu) (high-temperature oxidation zone) → (Cu) (primary-phase stability zone) → (Cu) + Cu_51_Zr_14_ (binary-phase decomposition zone) → (Cu) + Cu_51_Zr_14_ + Cu_4_Ti (ternary-phase decomposition zone). It is evident that the as-cast microstructure is observed in the low-temperature region, while numerous gray oxides are visible in the high-temperature region where the temperature reaches 820 °C. And pre-melt dendritic structures are observed between the (Cu) and Cu_51_Zr_14_ + (Cu) regions in Figure 3c. Note that in this work, the solid-state phase transformation occurred under diffusion-controlled conditions. According to the decomposition pathway, the primary phase is (Cu), with the subsequent precipitation of Cu_51_Zr_14_ and Cu_4_Ti, which is consistent with the solidification path reported in the literature [31] for alloy #63 (Cu-13.5Ti-3.5Zr, at.%). The difference is that Cu_51_Zr_14_ forms prior to Cu_4_Ti in this work, whereas during the solidification process, both Cu_51_Zr_14_ and Cu_4_Ti form simultaneously. One reason for this could be the higher thermal stability of Cu_51_Zr_14_ at high temperatures compared to Cu_4_Ti, which has a significant influence on the solid-state decomposition. Furthermore, the nucleation driving force and diffusion kinetics also play a significant role. Specifically, TDB data from Hermana et al. [23] indicate that at 820 °C, the nucleation driving force for Cu_51_Zr_14_ from the fcc-Cu matrix is –495.30 J/mol, while that for Cu_4_Ti is –14.84 J/mol. This substantial difference implies that Cu_51_Zr_14_ has a much greater tendency to nucleate under solid-state conditions. A larger driving force corresponds to a lower critical energy barrier for nucleation, favoring an earlier precipitation of Cu_51_Zr_14_. In addition, the diffusion kinetics further influence the phase evolution. During solid-state decomposition, the diffusion is relatively sluggish, and the impurity diffusion coefficient of Ti (5.032 × 10^−5^ m^2^/s) is lower than that of Zr (6.419 × 10^−5^ m^2^/s) in the Cu matrix [49]. Therefore, Cu_51_Zr_14_—which requires a higher Zr concentration—can nucleate and grow faster if local Zr supersaturation occurs early. In contrast, Cu_4_Ti nucleation may be delayed due to its lower driving force and the slower redistribution of Ti. By comparison, during solidification, rapid cooling allows for quick solute redistribution in the liquid, minimizing the thermal stability, nucleation driving force, and kinetic barrier in the solid phase. As a result, Cu_4_Ti and Cu_51_Zr_14_ can precipitate nearly simultaneously in the as-cast structure. In this work, the high-temperature microstructure is dominated by the (Cu) phase. This can be attributed to two factors: first, the thermal stability of (Cu) is higher than that of Cu_51_Zr_14_ and Cu_4_Ti; and second, the preferential oxidation or evaporation of Ti and Zr near 820 °C leads to local Cu enrichment in the high-temperature zone. 

**Figure 5:**
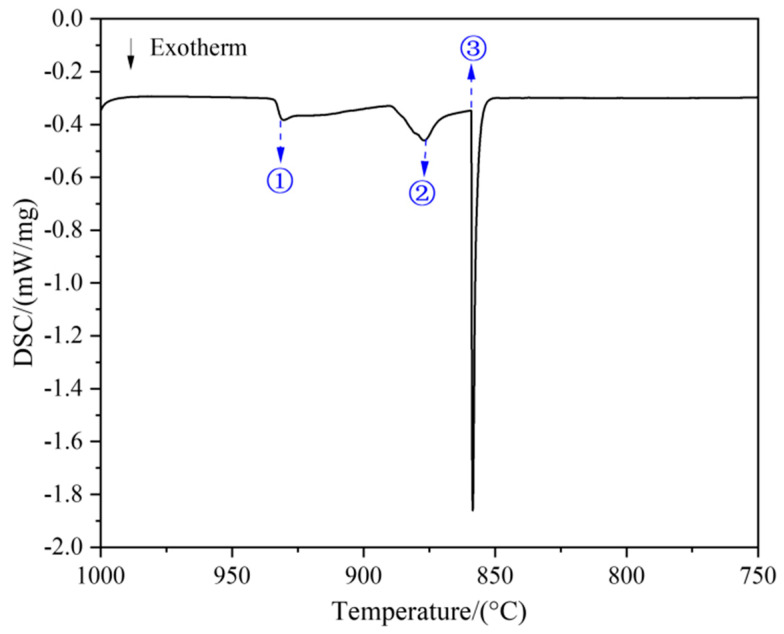
Cooling curve of the Cu-13.3Ti-3.8Zr (at.%) alloy.

Figure 5 illustrates the cooling curve of DSC for the as-cast region in the rod sample, covering the temperature range from 1000 to 750 °C, where two distinct exothermic peaks (① and ②) and one inflection point (③) are observed. Based on the previously analyzed solid-phase composition path, points ①, ②, and ③ are presumed to correspond to the phase transition temperatures of the Cu-13.3Ti-3.8Zr (at.%) alloy as follows: ① Liquid → Liquid + (Cu) at 930.4 °C; ② Liquid + (Cu) → Liquid + (Cu) + Cu_51_Zr_14_ at 876.1 °C; and ③ Liquid + (Cu) + Cu_51_Zr_14_ → (Cu) + Cu_51_Zr_14_ + Cu_4_Ti at 859.0 °C. When the temperature is higher than the phase equilibrium temperature (859.0 °C), the solid phase begins to transform into the liquid phase. However, as the induction coil temperature (820 °C) is below the phase equilibrium temperature, this indicates that the rod sample remained in a solid state during the heating process. Thus, the gravitational influence on the phase transition can be disregarded since no liquid phase was formed. Moreover, although the EPMA results revealed a gradual change in the solid-phase composition in the range of 800–820 °C, no distinct thermal signal was observed in the DSC curve (Figure 5). This is because the phase transformations occurring in this range are solid-state reactions involving limited atomic rearrangement and a relatively small enthalpy change. Such reactions typically result in a very weak heat flow that may fall below the detection limit of DSC equipment.

**Figure 6:**
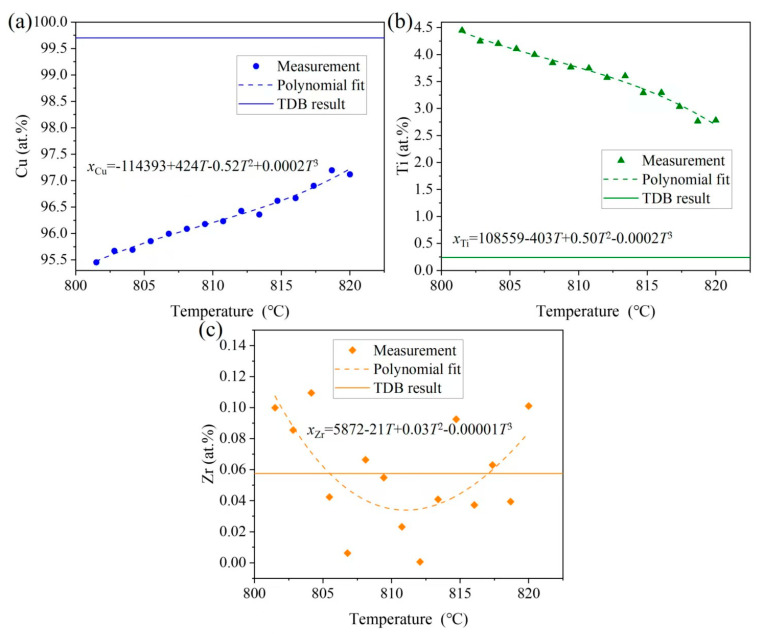
Comparisons of the TDB results from Hermana et al. [23] with measured and fitting results of the temperature–concentration gradient in the (Cu) solution of Layer 3: (**a**) Cu, (**b**) Ti, and (**c**) Zr. Note that the dotted curves represent the fitting results based on Equation (2).

The liquidus and solidus temperatures correspond to peaks ① and ③, respectively, which are close to a report in the literature [31] for the Cu-13.5Ti-3.5Zr alloy (at.%) after an aging treatment at 750 °C (liquidus: 903 °C, solidus: 879 °C), with deviations less than 30 °C. These differences can be attributed to supercooling effects in the cast alloy used in this work. When the temperature is higher than the solidus temperature (859.0 °C), the solid phase begins to transform into the liquid phase. However, as the temperature of the induction coil (820 °C) is below the solidus temperature, this indicates that the rod sample remained in a solid state during the heating process. Consequently, the gravitational influence on the phase transition can be disregarded since no liquid phase was formed. In Figure 3a, the primary-phase (Cu) zone (Layer 3) measured approximately 1.728 mm in length, and the heating induction coil is positioned on the right part, as shown by the red dotted line in Figure 3a. Noting that the temperature of the heating induction coil is 820 °C, with a temperature gradient of 10.7 °C/mm, the temperature range of this region can be estimated to be 820 °C → 801.5 °C. Subsequently, in the EPMA measurement, three parallel lines were drawn along the axis of the primary solidification zone (Layer 3), with 15 equidistant measuring points selected along each line for composition analysis. The average composition at points equidistant from the axis was calculated, yielding the composition distribution of the primary phase (Cu) within the temperature gradient. Figure 6 shows the distribution of Cu, Zr, and Ti contents in the primary phase (Cu) across the 820 °C to 801.5 °C temperature gradient. The data points are fitted using the following polynomial Equation (2):

(2) $$x_{i}=A+BT+CT^{2}+DT^{3}$$

where $x_{i}$ (*i* = Cu, Zr or Ti) is mole fraction of element *i*; A, B, C, and D are the fit parameters.

As shown in Figure 6, the Cu content in the solid solution of (Cu) increases with the increasing temperature from 95.45 to 97.12 at.%, while the Ti content decreases from 4.45 to 2.78 at.%. In contrast, the fluctuation in Zr content shows little correlation with the temperature, with an average solid solubility of 0.0575 at.%. Therefore, Figure 6 can be interpreted as representing the solid-state transformation boundary between (Cu) and (Cu) + Cu_51_Zr_14_ in the vertical section of Cu_0.999425_Zr_0.000575_/Cu_0.95_Ti_0.049425_Zr_0.000575_. The solid curves in Figure 6 correspond to the thermodynamic calculations by Hermana et al. [23] for this vertical section. The deviations between the experimental measurements and thermodynamic calculations [23] are less than 5 at.%, which falls within a reasonable margin of error. Here, 820 °C and 801.5 °C are located within the phase transformation temperatures from (Cu) to (Cu) + Cu_51_Zr_14_ of the compositions Cu_0.97017_Zr_0.00058_Ti_0.02925_ and Cu_0.95424_Zr_0.00058_Ti_0.04518_, respectively. 

### 3.2. DFT Calculation Results

**Figure 7:**
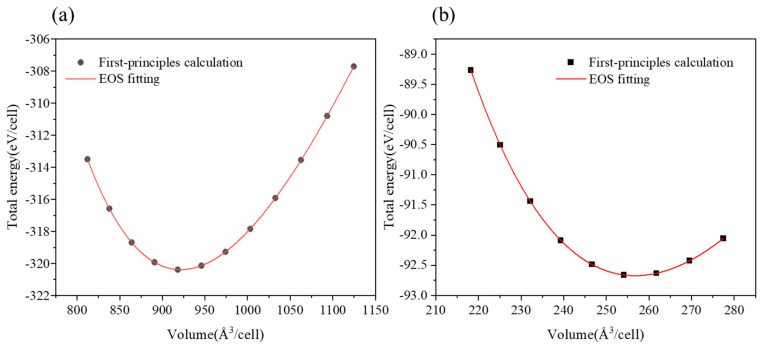
Relationship between total energy and volume in (**a**) Cu_51_Zr_14_ (**b**) Cu_4_Ti.

According to the experimental results (Table 1), the Cu_51_Zr_14_ compound can be doped with Ti in compositions ranging from 8.06 to 8.67 at.%. To investigate the thermal stability of the Cu_51_Zr_14_ compound with and without Ti doping, the formation enthalpies were estimated by first principle calculations. And the formation enthalpy of Cu_4_Ti was also estimated by first principle calculations. Firstly, the Birch–Murnaghan equation of state (EOS) was used for fitting to obtain the lowest energy for Cu_51_Zr_14_ and Cu_4_Ti. In this paper, a fourth-order Birch–Murnaghan (B-M) equation [50] is adopted to fit the energy (*E*) and cell volume (*V*) and is expressed as follows:

(3) $$E=a+bV^{-\frac{2}{3}}+cV^{-\frac{3}{4}}+dV^{-\frac{6}{3}}$$

where a, b, c, and d are fitting parameters. A set of energies (*E*) was obtained by varying the cell volume in steps of 0.01 from 0.96 to 1.04. The relationships between E and V for Cu_51_Zr_14_ and Cu_4_Ti were fitted using Equation (3), with the corresponding figure shown in Figure 7a and Figure 7b, respectively. The minimum energy and corresponding volume, estimated from the curve in Figure 7, define the stable structure for Cu_51_Zr_14_ and Cu_4_Ti. Next, based on the stable configuration, DFT calculations were conducted again with all crystal axes relaxed and the volume fixed. For example, the formation enthalpy of Cu_51_Zr_14_ ($H_{f}^{\mathrm{Cu}51\mathrm{Zr}14}$) was obtained using the following formula:

(4) $${∆H}_{f}^{\mathrm{Cu}51\mathrm{Zr}14}=(E_{\mathrm{tot}}^{\mathrm{Cu}51\mathrm{Zr}14}-xE_{\mathrm{fcc}}^{\mathrm{Cu}}-yE_{\mathrm{hcp}}^{\mathrm{Zr}})/N$$

where $E_{\mathrm{tot}}^{\mathrm{Cu}51\mathrm{Zr}14}$ is the total energy of Cu_51_Zr_14_ in a stable crystal structure; $E_{\mathrm{fcc}}^{\mathrm{Cu}}$ and $E_{\mathrm{hcp}}^{\mathrm{Zr}}$ are the ground-state chemical potentials (total energies per atom) for Cu and Zr, respectively; and N is the total number of atoms in the configuration.

For Cu_51_Zr_14_ with Ti doping, the EPMA analysis in Table 1 indicates an average composition of 77.73 at.% Cu, 13.91 at.% Zr, and 8.37 at.% Ti. Therefore, this compound can be approximately modeled as a Cu_51_Zr_9_Ti_5_ cell, in which five substitutional Ti atoms replace the Zr atoms in the Cu_51_Zr_14_ structure. The formation enthalpy of Cu_51_Zr_9_Ti_5_ ($H_{f}^{\mathrm{Cu}51\mathrm{Zr}9\mathrm{Ti}5}$) was obtained using the following formula:

(5) $${∆H}_{f}^{\mathrm{Cu}51\mathrm{Zr}9\mathrm{Ti}5}=(E_{\mathrm{tot}}^{\mathrm{Cu}51\mathrm{Zr}9\mathrm{Ti}5}-xE_{\mathrm{fcc}}^{\mathrm{Cu}}-yE_{\mathrm{hcp}}^{\mathrm{Zr}}-zE_{\mathrm{hcp}}^{\mathrm{Ti}})/N$$

where $E_{\mathrm{tot}}^{\mathrm{Cu}51\mathrm{Zr}9\mathrm{Ti}5}$ is the fully relaxed total energy of Cu_51_Zr_9_Ti_5_; $E_{\mathrm{hcp}}^{\mathrm{Ti}}$ is the ground-state chemical potentials (total energies per atom) for Ti.

**Figure 8:**
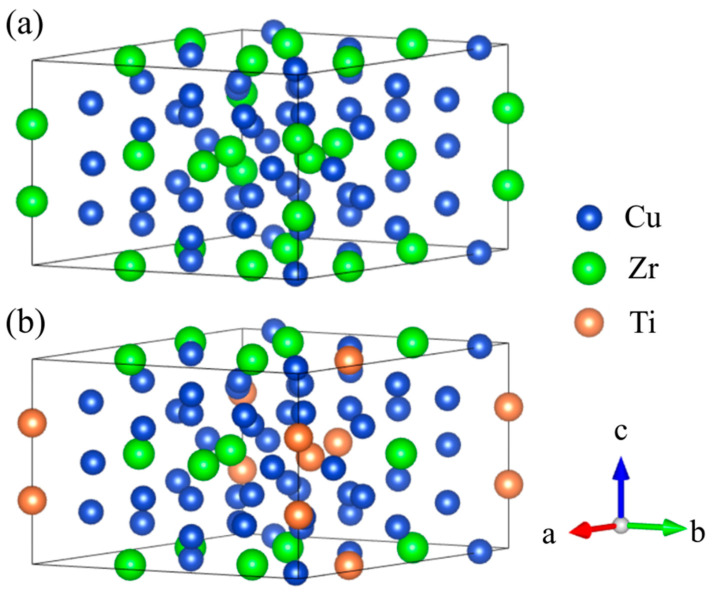
(**a**) Cu_51_Zr_14_ crystal structure, (**b**) Cu_51_Zr_9_Ti_5_ crystal structure.

**Figure 9:**
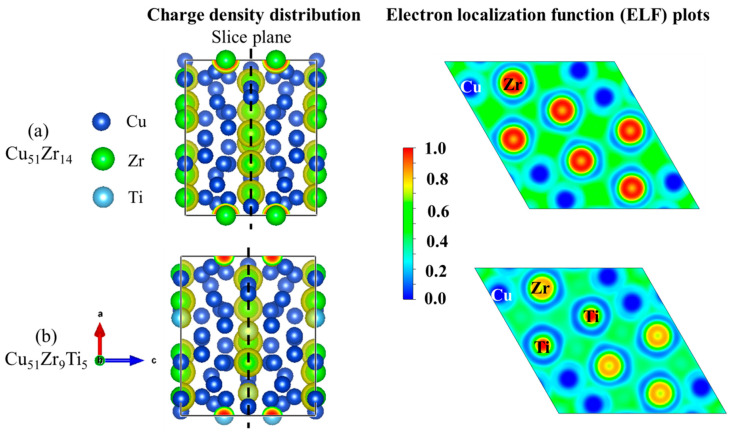
Charge density distribution and electron localization function of (**a**) Cu_51_Zr_14_ crystal structure and (**b**) Cu_51_Zr_9_Ti_5_ crystal structure.

Figure 8 illustrates the crystal structures of Cu_51_Zr_14_ and Cu_51_Zr_9_Ti_5_, and Table 2 compares the formation enthalpy of Cu_51_Zr_14_ and Cu_51_Zr_14_ with Ti doping using experiments at 298.15 K and first principle calculations at 0 K, respectively. Obviously, the formation enthalpy for Cu_51_Zr_14_ estimated in this work is 3.93 kJ/mol higher than the value obtained using the same PAW-GGA method reported by Zhou and Napolitano [51]. This deviation can be attributed to the absence of the EOS method step in Zhou and Napolitano [51]’s determination of the stable structure. Moreover, both the values from this work and those reported by Zhou and Napolitano [51] show significant deviation from the USPP-GGA method result provided by Ghosh [35]. The formation enthalpy of Cu_51_Zr_14_ obtained in this work is consistent with the result reported by experimental reports from Kleppa and Watanabe [52] and Weihs et al. [53] using a calorimeter as well as first principle calculations from Jain et al. [40], differing by less than 2.3 kJ/mol. The significant variation in the experimental formation enthalpy of Cu_51_Zr_14_ reported in different studies is likely attributable to differences in the purity of raw materials and the heating rate employed. Based on first principle calculations, the formation enthalpy of Cu_51_Zr_9_Ti_5_ is –13.44 kJ/mol, which is 2.87 kJ/mol higher than that of Cu_51_Zr_14_ (–16.31 kJ/mol), indicating the thermal stability of the Cu_51_Zr_14_ compound deteriorates after Ti doping in the Cu-Ti-Zr system. For Cu_4_Ti with the space group *Pnma*, the formation enthalpy obtained in this work agrees well with values reported in the literature [36,40,54], with a deviation of less than 2 kJ/mol. The formation enthalpy of Cu_4_Ti estimated in this work is –9.57 kJ/mol, which is 3.87 kJ/mol higher than that of Cu_51_Zr_9_Ti_5_. This indicates that Cu_51_Zr_9_Ti_5_ exhibits superior thermal stability compared to Cu_4_Ti, consistent with the results observed in the temperature gradient experiment. Figure 9 shows the charge density distribution and electron localization function (ELF) of Cu_51_Zr_14_ and Cu_51_Zr_9_Ti_5_. As shown in Figure 9a, Cu_51_Zr_14_ exhibits strong electron localization around Zr atoms, suggesting pronounced directional bonding. In contrast, as shown in Figure 9b, with the partial substitution of Zr with Ti (Cu_51_Zr_9_Ti_5_), the charge distribution becomes more uniform, and the ELF values around both the Zr and Ti atoms decrease significantly. This increased electronic delocalization reduces internal stress concentrations, facilitates atomic mobility, and contributes to improved structural and electrical stability.

## 4. Conclusions

We have provided a hybrid approach involving a temperature–concentration gradient and DSC analysis to determine the solid-state thermal decomposition pathway in the Cu-13.3Ti-3.8Zr (at.%) alloy. The primary phase is (Cu), followed by the precipitation of the Cu_51_Zr_14_ and Cu_4_Ti phases, which is consistent with the solidification pathway reported in the literature. The solution of Cu, Ti, and Zr in the (Cu) phase was determined along the temperature gradient, and this method can be further applied to phase diagram research, significantly reducing the time required compared to the traditional phase equilibrium alloy method. Furthermore, based on the experimental results, DFT calculations were performed to analyze the effect of Ti doping on the Cu_51_Zr_14_ compound. The main conclusions are as follows:

(1) The solid-state thermal decomposition pathway of the Cu-13.3Ti-3.8Zr (at.%) alloy proceeds as follows: (Cu) → (Cu) + Cu_51_Zr_14_ → Cu_51_Zr_14_ + (Cu) + Cu_4_Ti, and the liquidus and solidus temperatures are 930.4 °C and 859.0 °C, respectively;

(2) The solid solubility of Cu, Ti, and Zr in the primary phase (Cu) in the temperature range of 820 to 801.5 °C is determined. As temperature increase, the Cu content increases from 95.45 to 97.12 at.%, the Ti content decreases from 4.45 to 2.78 at.%, and the Zr content remains relatively constant with an average solid solubility of 0.0575 at.%;

(3) The EPMA and XRD analysis show that the average composition of the Cu_51_Zr_14_ compound in the Cu-Ti-Zr system is 77.73 at.% Cu, 13.91 at.% Zr, and 8.37 at.% Ti. The DFT calculations indicate that substituting Ti for Zr in Cu_51_Zr_14_ increases the formation enthalpy of Cu_51_Zr_9_Ti_5_ to –13.44 kJ/mol, compared to –16.31 kJ/mol for Cu_51_Zr_14_, suggesting a decrease in thermal stability due to Ti doping. The formation enthalpy of Cu_4_Ti is –9.57 kJ/mol, which is higher than that of Cu_51_Zr_9_Ti_5_, indicating that Cu_51_Zr_9_Ti_5_ exhibits a superior thermal stability compared to Cu_4_Ti. Moreover, the charge density distribution and ELF analysis reveal that Ti doping can enhance the structural and electrical stability of the Cu_51_Zr_14_ compound.

## Figures and Tables

**Table 1:** Constituent phases and corresponding compositions of various regions in the Cu-13.3Ti-3.8Zr (at.%) alloy.

Area	Phases	Composition (at.%), EPMA		
		Cu	Zr	Ti
Figure 3b	Cu_4_Ti	79.20 ± 3.36	1.42 ± 0.52	19.38 ± 2.66
	Cu_51_Zr_14_	76.67 ± 1.02	14.66 ± 2.31	8.67 ± 3.37
	(Cu)	93.62 ± 3.23	0.01 ± 1.65	6.37 ± 3.90
Figure 3c	Cu_51_Zr_14_	78.79 ± 0.13	13.15 ± 0.24	8.06 ± 0.27
	(Cu)	94.78 ± 3.63	0.06 ± 1.48	5.16 ± 1.74
Figure 3d	Oxidation zone	3.45–33.26	1.35–81.87	0.79–67.19
	(Cu)	97.08 ± 0.74	0.31 ± 1.10	2.61 ± 0.72

Note: Compositions were measured by EPMA. Uncertainties (±) indicate the 95% confidence intervals from multiple measurements. Ranges indicate composition fluctuations across inhomogeneous regions.

**Table 2:** Formation enthalpies of Cu_51_Zr_14_, Cu_51_Zr_9_Ti_5_, and Cu_4_Ti from experiments and first principle calculations, respectively.

Phase (Space Group)	Method	${∆}_{f}H$, (kJ/mol-atom)	Temperature	DFT Details	Reference
Cu_51_Zr_14_ (*P6*)	Experimental (Calorimeter)	–14.07 ± 1.24	298.15 K	–	[52]
	Experimental (Calorimeter)	–14.38 ± 0.3	298.15 K	–	[53]
	Experimental (Knudsen effusion mass spectrometry)	–11.241 ± 0.076	298.15 K	–	[55]
	Experimental (Calorimeter)	–24.30 ± 2.2	298.15 K	–	[56]
	Experimental (Calorimeter)	–25.20	298.15 K	–	[57]
	DFT	–20.24	0 K	PAW-GGA	[51]
	DFT	–8.64	0 K	USPP-GGA	[35]
	DFT	–15.73	0 K	PAW-GGA	[40]
	DFT	–16.31	0 K	PAW-GGA	This work
Cu_51_Zr_9_Ti_5_ (*P6*)	DFT	–13.44	0 K	PAW-GGA	This work
Cu_4_Ti (*Pnma*)	Experimental (Thermography)	–9.593	298.15 K	–	[54]
	DFT	–7.90	0 K	PAW-GGA	[36]
	DFT	–8.10	0 K	PAW-GGA	[40]
	DFT	–9.57	0 K	PAW-GGA	This work

## Data Availability

The original contributions presented in this study are included in the article. Further inquiries can be directed to the corresponding authors.
